# Old Fellows

**Published:** 1871-08

**Authors:** 


### OLD FELLOWS.

Within the last twenty years a good many old codgers, bordering on the nineties, have been seized with the desire of informing the public how they live, what they eat, the duration of their sleep, their modes of spending their time, and a thousand and one particulars about matters more or less important; but all go to the effect of befogging the general reader, and leave him at a greater loss than ever as to the important point, " How am I to live so as to reach a healthful old age ? " because no two agree in any half dozen things. Grant Thorburn, at ninety, drank a quart or two of strong coffee at every meal; we really should think that was enough to kill any common man. Another, away out West who had passed his hundred, claimed to have lived mainly on corn bread and bacon ; yet there are multitudes besides the Hebrews who seriously believe that pork is poison. Within a week the announcement is made that a fellow beyond the Mississippi, now ninety years old, and who is as lively as a boy, and full of fun, attributes his joyous old age to the fact that every morning since his youth he has scrubbed his hide with a corn-cob; perhaps if he had scrubbed it with two, or repeated the operation with one every night, he might have lived to a hundred and eighty. The latest blast is from the trumpet of a poet; his greatest forte is monkey capers; he spends an hour every day in beating the air, in imaginary fisticuffs; at one time he takes up his chair and swings it around his head with amazing velocity; at another he catches hold of a wooden bar a foot or two above his head, and experiments on his toeing the ceiling. For variety, we suggest that he put himself on the floor, then get a friend to lock one leg around the neck, then leave him to get it back himself. He is great, too, on bathing; he can't eat his breakfast until he gets his bath; but he seems to live in spite of that dangerous ordeal — dangerous always to the young, the old, and the feeble. A great point, insisted upon with apparent self-commendation, is that he never takes stimulants; he abhors lager, eschews Bourbon, and regards Cogniac and Champagne as " things that are vain." We saw an old fellow in the cars the other day with as bright an eye as we have seen for a long time, unexceptionally neat in his apparel, with the courtly manners of a gentleman of the olden time. He eats enough opium every day to kill five men outright, and is one hundred and five years old.

We are not to secure a healthy old age by doing one thing, or by avoiding another; it is arrived at in all cases by a combination of circumstances, and of a favoring character. But there are a few cardinal points which best insure the desired result. Be just, live temperately, regularly, industriously, and maintain an habitual equanimity.

